# Adenovirus serotype 26 utilizes CD46 as primary cellular receptor and only transiently activates T lymphocytes following vaccination of rhesus monkeys

**DOI:** 10.1186/1742-4690-9-S2-O69

**Published:** 2012-09-13

**Authors:** H Li, EG Rhee, K Masek-Hammerman, JE Teigler, P Abbink, DH Barouch

**Affiliations:** 1 Beth Israel Deaconess Medical Center, Boston, MA, USA; 2 New England Primate Research Center, Southborough, MA, USA

## Background

Adenovirus serotype 5 (Ad5) utilizes coxsackievirus and adenovirus receptor (CAR) as its primary cellular receptor. However, the cellular receptor utilized by Ad26 and the inflammatory responses elicited following Ad26 vaccination remain unclear.

## Methods

Receptor usage was assessed using CD46 transgenic mouse cells, as well as by CAR- and CD46-specific mAb blocking studies using human PBMC. Twelve adult rhesus monkeys were inoculated with of 10^11^ viral particles (vp) of replication-competent Ad5 and Ad26 (N=6) or saline (N=6) at weeks -8 and -4, and were vaccinated intramuscularly with 3×10^10^ vp replication-incompetent Ad26-Gag/Pol/Env vectors. At week 2, monkeys were sacrificed to assess immunologic and inflammatory responses at mucosal surfaces.

## Results

Transduction by Ad26 and Ad35 vectors was markedly enhanced in CD46 transgenic mouse cells compared with wild type mouse cells. Moreover, transduction of human PBMC by Ad26 and Ad35 vectors was efficiently blocked by the anti-CD46 mAbs 13/42, M177 and MEM-258, but not by the anti-CAR mAbs RmcB and E1-1. Monkeys with and without baseline Ad5/Ad26 immunity exhibited similar magnitude and only transient activation (1-2 weeks) of vector-specific CD4^+^ T cell responses in both PBMC and colorectal biopsies. Inflammatory cell infiltrates in colorectal and foreskin mucosa were comparable in baseline and vaccinated animals regardless of baseline Ad5/Ad26 immunity.

## Conclusion

Ad26 utilizes CD46 and not CAR as a primary cellular receptor for infection. We also observed no increased mucosal cellular activation or vector-specific CD4^+^ T lymphocytes in baseline Ad5/Ad26-seropositive monkeys as compared with baseline seronegative monkeys following Ad26 vaccination. These data contribute to our understanding of the biology of Ad26 as a candidate vaccine vector.

